# Reduction in mental distress among substance users receiving inpatient treatment

**DOI:** 10.1186/1752-4458-4-30

**Published:** 2010-12-02

**Authors:** Ellen Hoxmark, Vår Benum, Oddgeir Friborg, Rolf Wynn

**Affiliations:** 1 Department of Substance Use and Specialized Psychiatric Services, University Hospital of Northern Norway, 9291 Tromsø, Norway; 2 Department of Clinical Medicine (FT), University of Tromsø, 9037 Tromsø, Norway; 3 Department of Psychology, University of Tromsø, 9037 Tromsø, Norway; 4 Psychiatric Research Centre of Northern Norway, University Hospital of Northern Norway, 9291 Tromsø, Norway

## Abstract

**Background:**

Substance users being admitted to inpatient treatment experience a high level of mental distress. In this study we explored changes in mental distress during treatment.

**Methods:**

Mental distress, as measured by the HSCL-10, was registered at admission and at discharge among 164 substance users in inpatient treatment in Northern Norway. Predictors of reduction in mental distress were examined utilizing hierarchical regression analysis.

**Results:**

We found a significant reduction in mental distress in the sample, but the number of patients scoring above cut-off on the HSCL-10 at discharge was still much higher than in the general population. A more severe use of substances as measured by the AUDIT and the DUDIT, and being female, predicted a higher level of mental distress at admission to treatment as well as greater reduction in mental distress during treatment. Holding no education beyond 10 year compulsory school only predicted a reduction in mental distress.

**Conclusions:**

The toxic and withdrawal effects of substances, level of education as well as gender, contributed to the differences in change in mental distress during treatment. Regression to the mean may in part explain some of the findings.

## Background

In studies of the general population, the incidence of mental disorders among people with substance use disorders (SUD) varies according to catchment area and methodology. It is assumed that 30 - 40% of people with alcohol related disorders, and 40 - 50% of people with other SUD, also have a psychiatric disorder [1-5]. The incidence of psychiatric disorders among individuals with SUD in treatment is even higher [6,7]. Psychiatric disorders have repeatedly been shown to influence treatment outcomes in different treatment seeking SUD samples [8-10].

The psychiatric disorders most commonly associated with SUD are anxiety and depression [11-13]. Symptoms of anxiety and depression have been found to influence the course of treatment [10], and to predict relapse in SUD [14-16]. On the other hand, several studies have found that anxiety and depressive symptoms among SUD patients often are passing, representing toxic or withdrawal effects that resolve in response to abstinence or to entry into SUD treatment [17-21]. Longitudinal studies in representative population samples suggest that casual relationships can operate in various directions between SUD and symptoms of anxiety and depression [22]. Anxiety and depression could increase the likelihood of developing SUD; the development of SUD among those with anxiety and depressive symptoms could worsen their course, and symptoms of anxiety and depression could reflect substance-induced conditions [23]. Previous studies of SUD treatment have shown that patients who stay in treatment longer more likely achieve the best outcomes, regardless of outcome measures [20,24].

In screening for psychiatric disorders, the concept of mental distress is widely used. One screening instrument used in population studies is the HSCL-10 [25]. The HSCL-10 has been developed from the original HSCL-90 [26], using two (i.e. anxiety and depression) out of the nine original dimensions [27]. In the general population, it has been found that 11.4% of the population meets the criteria for further assessment and treatment of psychiatric disorders [25]. In one study, it was concluded that 50 - 60% of the "cases" identified by instruments like the HSCL-10 meet the criteria for a diagnosis of a psychiatric disorder [28]. Prior studies have described a close relation between substance use and mental distress [29,30].

Epidemiological surveys have consistently documented higher rates of anxiety and depression among women than men [31,32], and it is a common finding that women report a higher level of mental distress [33]. In a treatment seeking sample of patients with SUD in Norway, women more often suffered from depression than men [6]. In a follow-up of the same sample six years after treatment, Bakken et al. [34] found that mental distress remained high at the time of follow-up, and that abstainers had a significantly lower level of mental distress, especially female abstainers. This finding is consistent with other studies that have shown that women in SUD treatment generally report a higher level of mental distress [35].

In the general population, higher education is known as a protective factor with respect to physical and mental health [36,37]. A Norwegian population study concluded that a higher level of education seemed to have a protective effect against anxiety and depression [38]. In Norway, more than 80% of the population hold an education beyond 10 year compulsory school [39]. There is a consistent relationship between dropping out of high school and substance use [40]. In one study, it was found that drop-outs used substances at elevated levels compared with in-school peers, and the drop-outs were more likely to develop alcohol related disorders [41]. In another study, drop-out from school in Norway was shown to be related to frequent alcohol intoxications [42]. Typically, those in lower socio-economic groups have worse health and higher mortality than those in higher socio-economic groups [43,44]. SUD and other psychiatric disorders are generally associated with a variety of psychosocial risk factors [45-48].

In a recent study [30], we examined predictors of mental distress at admission to in-patient SUD treatment. This study indicated that gender had a significant impact on the level of mental distress, as women scored higher on mental distress at admission to treatment. A more severe use of substances, as reported on the AUDIT [49] and the DUDIT [50], also predicted a higher level of mental distress, as well as having previously received psychiatric treatment. In this study, 83% of the patients scored above the established cut-off for the HSCL-10 at admission.

The aim of the present study was to determine predictors of change in the level of mental distress among SUD patients in inpatient treatment. First, we examined to which extent mental distress changed during treatment. Second, we examined possible determinants of the change in mental distress.

## Material and methods

This is the first study on substance use, mental distress and treatment outcomes in Northern Norway including patients in several units. The project was based on a naturalistic design with measurements taken before treatment and at discharge. All patients admitted to the units and considered competent to consent during the period September 2007 to May 2009 were given written and oral information about the study by a research collaborator working in each unit.

### Material

Data was collected from 164 patients admitted to one of the five inpatient treatment units for SUD use within the catchment area of the University Hospital of Northern Norway. In the study period, 574 patients were admitted to the units. Patients who were considered not able to give an informed consent (*N *= 21) or whose hospital stay was too short too be included (*N *= 41) were not asked to participate. Of the patients considered relevant for the study (*N *= 512), 296 patients (58%) agreed to participate and signed an informed consent. Of these, 273 patients filled out the questionnaire at admission, and of these 172 filled out the questionnaire at discharge. Patients (*N *= 8), who had failed to complete the HSCL-10 in both of the questionnaires, were excluded from the analyses. None of the patients' replies deviated strongly statistically. The final sample thus consisted of 164 respondents (74% men, mean age 40, range 18 - 67 years). More than 90% of the sample had a Norwegian origin.

As only two percent of patients from the catchment area were referred to outpatient treatment in 2005 [51], the inpatient group was highly representative of all patients. The units covered a population of 500 000 inhabitants in the counties of Nordland, Troms and Finnmark. Unit 1 offered specialized assessment and treatment of dual diagnoses and provided treatment up to six months (*N *= 12) Unit 2 provided treatment up to 18 months according to a therapeutic community model (*N *= 9). Unit 3 was a detoxification unit that provided treatment up to six weeks (*N *= 54). Unit 4 and 5 provided general SUD inpatient treatment up to six months (*N *= 43 and *N *= 48). All units treated both sexes, used a combination of group and individual therapy, and managed detoxification directly (Unit 2 and 3) or in collaboration with a detoxification unit (Unit 1, 4 and 5). Treatment was composed of a combination of network-based approaches, psychotherapeutic and pharmacological treatments, but these components were given different emphasis in the various units.

### Instruments

#### Outcome

##### Mental distress

Mental distress was measured twice, before admission and at discharge, using a 10 item version of the Hopkins Symptom Check-List (HSCL-10) [25]. The HSCL-10 is a self-report questionnaire with a four point Likert scale, ranging from 1 (not at all) to 4 (extremely). The HSCL-10 is based on the SCL-90-R [26], and is composed of two out of the original nine factors (anxiety and depression) [27]. A mean item score was calculated and used as an index of general distress severity. Missing data were replaced with the mean value if no more than two item scores were missing [52]. An average score of 1.85 or higher indicates a need of further assessment and possibly a need for psychiatric treatment [25].

##### Improvement in mental distress during treatment

Improvement was measured as a reduction in the mean HSCL-10 scores from pre to post. Change scores were calculated by subtracting the mean post score from the mean pre score. As the purpose of using change scores in the present study was to identify predictors of change, rather than evaluating absolute change, the reliability of change scores may be estimated with Cronbach's method [53]. The internal consistency of the change scores was very good (α = .84), while the reliability of the pre- and post-test scores were comparable (α = .91).

#### Predictors

##### Alcohol and drug use

Substance use was measured by the Alcohol Use Disorders Identification Test (AUDIT) [54] and the Drug Use Disorders Identification Test (DUDIT) [55]. The AUDIT is a widespread instrument measuring severity of alcohol use the past 12 months. It has 10 items with a scoring range from 0 to 40. The DUDIT is a parallel instrument to the AUDIT and is designed to identify persons with drug use problems the past 12 months. It has 11 items with a scoring range from 0 to 44.

Current use of specific substances were measured by the self-report Drug Use Disorders identification Test - Extended (DUDIT-E) [56]. Responses to the DUDIT-E were coded 0 if a substance was used less than twice a month, and 1 if used twice a month or more often [57]. The number of substances used was calculated from the number of substances used twice a month or more as reported on the DUDIT-E, together with alcohol used twice a week or more as reported on the AUDIT.

##### Variables related to admission and discharge

The patients' socio-demographic and treatment histories were assessed using the Norwegian National Client Assessment Form [58]. Variables in this form include age, sex, occupation, housing, and previous treatment. This form is routinely completed for all patients admitted to SUD treatment in Norway. In addition, the patients' clinicians provided extended information on the patients' socio-demographic history and diagnostic assessment through a form developed especially for this study.

The patients stayed in treatment for an average of 56 days (range 3 - 396). The variable defining the treatment setting was dichotomized into the detoxification unit (*N *= 54) and all other units (*N *= 110).

The kind of treatment offered to the patients was registered on a form listing 18 possible treatments, e.g. individual treatment by a clinical psychologist, psychotropic medication, group treatment and family counseling.

### Procedures

Shortly following their consent to participate they responded to the questionnaire. Before discharge, patients responded to a second questionnaire including corresponding questions, as well as questions about treatment satisfaction. Patients were paid compensation in the form of a cinema ticket or two lottery tickets (worth $8). The study was approved by the Regional Committee for Medical Research Ethics (P REK Nord 12/2006) and the Norwegian Social Science Data Services (NSD).

### Statistical Methods

All analyses were conducted using SPSS 16.0. Internal consistency of test scores were assessed with Cronbach's alpha [53]. A hierarchical regression analysis was conducted to identify predictors of HSCL-10 change scores [59]. The predictors were entered in the regression model in three steps: 1) demographic variables (age, gender, employment, marital status, living conditions, and education), 2) substance use (score on AUDIT and DUDIT, number of substances used), and 3) treatment variables (previous substance use or psychiatric treatment, number of days in treatment, treatment in the detoxification unit (Unit 3), psychotropic medical treatment, and treatment by clinical psychologist). As we did not have a clear theory or other empirical studies done in Norway to guide the selection of variables within each cluster (block) of predictors, a stepwise procedure was used. Regression diagnostics were performed to test for collinearity, normality, outliers, and leverage. Effect size statistics were reported as Cohen's *d *(paired *t*-tests). According to Cohen [60], effect sizes of .2, .5 and .8 represent weak, moderate and strong effects, respectively.

## Results

The most frequently occurring substance use problem, according to diagnoses reported by the clinicians, was alcohol dependence (60%), opiate dependence (29%), dependence to hypnotics (20%), and amphetamine dependence (19%). Concurrent polydrug use was common as 64% reported using two or more substances, and 42% using three or more substances.

### Reduction in Mental Distress

On average, the participating patients reported a high mean score of mental distress at admission (*M *= 2.54, *SD *= .75), which decreased during treatment, being significantly lower (*M *= 1.86, *SD *= .67) at discharge (*t*_163 _= 13.24, *p *< 0.001). The statistical effect size of this reduction was very strong (*d *= 1.14). The proportion of patients scoring above the established cut-off-level of 1.85 on HSCL-10 fell from 82% at admission to 44% at discharge.

### Predictors of change in Mental Distress

The mean and the standard deviation of mental distress change scores were -.68 and .66, respectively. Among the 164 patients, 141 patients reported a reduction in distress (max positive change: -2.80), while 4 patients reported no change and 19 patients reported a negative change by experiencing more mental distress at discharge (max negative change: 1.30). In order to identify which factors that were related to improvement during treatment, a hierarchical regression analysis was conducted on the change scores in mental distress. The standardized regression coefficients are presented in Table 1. Demographic variables were entered into the first step. Two of the six variables contributed significantly. Education explained 3.1% of the variance (*Adj R^2^*) (*F*_1, 162 _= 6.21, *p *= 0.014), indicating that having no education exceeding 10 year compulsory school, was related to a larger reduction in HSCL-scores. Gender explained an additional 2.6% of the variance (*F-*change_1, 161 _= 4.48, *p *= 0.036), indicating that women experienced a larger reduction in HSCL-10 scores. In the second step three substance use variables were entered, and two of these contributed significantly. Variation in the AUDIT scores explained an additional 3.0% of the variance (*F*-change_1, 160 _= 5.47, *p *= 0.021), and variation in the DUDIT scores explained an additional 4.3% of the variance (*F-*change_1, 159 _= 7.75, *p *= 0.006), indicating that a more severe use of alcohol and other substances was related to more reduction in HSCL-10 scores. In the third step, none of the variables related to type of treatment contributed significantly. The final regression coefficient parameters with HSCL-10 change scores as the criterion variable are shown in Table 1.

**Table 1:** Stepwise hierarchical regression analysis predicting change in HSCL-10 scores (N = 164)

Step and predictor	*ß*	*R^2^*	*ΔR^2^*	*Final ß*
Step 1		.06	.06*	
Education (1 = post compulsory school)	-0.19*			-0.21**
Gender (1 = men)	-0.16*			-0.17*
Step 2		0.11	0.07*	
DUDIT	0.21**			0.28**
AUDIT	0.19*			0.19*

*Note*. * *p *< .05, ** *p *< .01.

## Discussion

The present study demonstrated that mental distress changed significantly during treatment for patients admitted to inpatient SUD treatment. A lower level of education, being female and having a more severe use of substances, all predicted a greater change in mental distress during treatment. Being female and having a more severe use of substances were also connected to a higher level of mental distress at admission to treatment [30].

Even though change in mental distress from admission to discharge was substantial in this sample, the level of mental distress was still high at discharge. Almost half of the sample scored above cut-off on the HSCL-10, as opposed to 11.4% in the general population in Norway [25]. Regression to the mean may in part explain some of the findings. It is a well known fact that the people that have the highest scores (or the most problems) also are in the position to change most. Despite this, a score of change like the one that is used in this study has been shown to be highly reliable [61].

A more severe use of substances predicted a larger reduction in mental distress during treatment. This could mean that mental distress is connected to the use of substances, and that these symptoms decrease as use of substances decrease and symptoms of intoxication and withdrawal vanish. This assumption is supported by previous studies which have shown that psychiatric symptoms like depression and anxiety to a great extent change after some time in treatment [17,21]. Moreover, some depressive symptoms resolve rapidly after brief periods of abstinence or entry into SUD treatment [18-20].

To some extent, symptoms of depression and anxiety among SUD patients can be seen as toxic or withdrawal symptoms, and can therefore be expected to vanish during treatment. Still, the proportion of patients with a mental distress score above cut-off at discharge is high - 44%. These findings are consistent with previous studies of SUD treatment, which have shown that patients who stay in treatment longer more likely achieve the best outcomes, regardless of the outcome measure [20,24]. This finding could be an argument for offering treatment that is extended beyond the withdrawal phase.

Women reported a significantly larger reduction in mental distress during treatment than men. As we found in a prior study, there was also a significant difference in the level of mental distress between men and women at admission to treatment [30]. It is a well known fact from population studies that women report a higher level of mental distress and qualify more often for diagnoses of anxiety and depression [32,33]. The finding from our study is consistent with prior results [6,34], suggesting that women experience a higher degree of mental distress and also a greater reduction in mental distress if abstinent. This result could also reflect the fact that SUD treatment in our area is more adjusted to the needs of women.

A possibly surprising result in this study was that a lower level of education (i.e. 10 year compulsory school at the most) was associated with a greater change in mental distress during treatment. Population studies have found that those in lower socio-economic groups in general have worse health and higher mortality than those in higher socio-economic groups [43], and in the general population the prevalence of mental distress has been found to increase by decreasing social status [44]. A higher education level has been found to have a protective effect against anxiety and depression [38]. Education is known as a factor that impacts physical health, in the way that education may act to support positive lifestyle choices and the development of habits that over time maintain physical health [37]. A number of psychosocial factors have been shown to be related to the development of problematic substance use [46]. In our study, it was only the difference between no education beyond compulsory school and any further education that was significant. 43% of the sample was in the group that had no education beyond compulsory school. This is a higher percentage than in the general Norwegian population, where only 21% have no education beyond compulsory school [39]. An early onset of SUD is related to school drop-out [40-42]. The fact that patients with less education experienced significantly more reduction in mental distress than those with more education could reflect that developing a SUD causes a downward shift in socio-economic group [48], and in this sense implies a greater loss of functions, a higher sense of loss, and a greater degree of stigma for people with more education than those with less. Further research is necessary to understand this phenomenon, for instance by conducting qualitative interviews of patients, focusing on the content of treatment.

### Strengths and limitations

The present study is subject to a number of limitations. The study sample was selected from five different units for inpatient SUD treatment. The units differed substantially - one unit was primarily concerned with detoxification, one unit focused on the assessment of dual diagnosis patients, and three units offered a more goal directed SUD treatment. There is, therefore, some heterogeneity within the sample. The participation rate was also relatively low. A proportion of the original participants did not complete the survey at discharge. A potential drawback with calculating change scores is the risk of a marked reduction in test score reliability as measurement errors at two points in time are added together, hence increasing total measurement error. The consequence is loss of statistical power. However, a substantial reduced reliability is not always the case, as was demonstrated in a study using generalizability theory to estimate absolute and relative reliability of change scores [61]. If the variance in change scores and the number of items are large enough, the reliability of change scores may be adequately high. A further limitation can be that the study lacks an untreated control condition, although this is extremely difficult to construct in this study setting. On the other hand, multisite, prospective studies like the present can investigate treatment outcomes in existing services and under actual clinical circumstances, and thereby show a high external validity and allow for a generalization of the findings to clinical settings [20].

## Conclusions

Patients with SUD admitted to inpatient treatment reported a significant reduction in mental distress through treatment. An increased severity in the use of substances, as well as being female, both predicted a higher level of mental distress at admission to treatment and a greater change in mental distress during treatment. Holding no education beyond compulsory school only predicted a reduction in mental distress during treatment. The toxic and withdrawal effects of substances, level of education as well as gender, probably explain most of the differences in change. Some of these changes may in part be explained by regression to the mean.

## Competing interests

The authors declare that they have no competing interests.

## Authors' contributions

EH participated in designing the study, collecting the data, analysing and interpreting the data, and in drafting and revising the manuscript. VB participated in designing the study, interpreting the data, and drafting and revising the manuscript. OF participated in interpreting the data, and drafting and revising the manuscript. RW participated in designing the study, analysing and interpreting the data, and in drafting and revising the manuscript. All authors read and approved the final manuscript.
